# Climate Change Drives Divergent Potential Habitat Dynamics of Invasive and Native Noxious Asteraceae Weeds in Yunnan Grasslands

**DOI:** 10.3390/plants15081217

**Published:** 2026-04-16

**Authors:** Jianglongze Yang, Peng Chen

**Affiliations:** 1 College of Forestry, Southwest Forestry University, Kunming 650224, China; 15925560812@163.com; 2 Yunnan Academy of Forestry and Grassland, Kunming 650201, China

**Keywords:** MaxEnt model, invasive vs. native species comparison, climate change, habitat suitability prediction, Yunnan province

## Abstract

Using high-resolution field data from the Yunnan Provincial Grassland Pest Survey and an optimized MaxEnt model, we compared the climate-driven habitat dynamics of two invasive Asteraceae weeds (*Chromolaena odorata*, *Ageratina adenophora*) and a native weed (*Cirsium japonicum*). We assessed whether invasive and native weeds differ in environmental responses, future range dynamics, and management strategies, and three novel patterns were revealed. First, the invasive *Chromolaena odorata* exhibits a sustained positive response to mean annual temperature (contribution 67.6%), while the native *Cirsium japonicum* shows a strictly unimodal response with a narrow optimum (0–10 °C, contribution 46.4%) and high-temperature sensitivity, projecting over 50% habitat loss by the 2050s under high emissions. Second, the invasive *Ageratina adenophora* displays a southern contraction versus northern expansion pattern under high emissions (current highly suitable area ~9.12 × 10^4^ km^2^), suggesting that extreme warming may enable it to breach high-altitude barriers. Third, all three species show unimodal responses to human disturbance with species-specific optima. Overall, the invasive species, leveraging broad ecological amplitudes and strong adaptability, are poised for continued expansion of their potential suitable habitat, while the native species, constrained by a narrow niche and limited dispersal capacity, faces systemic habitat loss. These findings provide a mechanistic basis for differentiating management strategies between invasive and native problematic weeds in Yunnan grasslands.

## 1. Introduction

Yunnan Province is rich in grassland resources and constitutes an important component of southern Chinese grasslands, which are primarily distributed in central, southern, northwestern, and northeastern Yunnan [1]. The province has a total natural grassland area of approximately ~134,630 km^2^, accounting for 40.3% of its land area, of which the usable grassland area reaches ~104,820 km^2^. The grassland types are mainly thermo-therophytic shrub–grassland, warm-temperate therophytic shrub–grassland, and mountain meadow, exhibiting a gradual transition from south to north and from low to high altitude [2]. Influenced by natural conditions and human activities, grassland degradation is prevalent in Yunnan, with moderately and lightly degraded grasslands accounting for about 60% and severely degraded grasslands accounting for approximately 20% [3].

The proliferation and spread of noxious weeds during grassland degradation negatively affect ecosystems, becoming a key factor constraining sustainable grassland utilization [4]. According to surveys, the distribution area of noxious weeds in the province’s natural grasslands reaches ~29,333 km^2^, accounting for 24.71% of the usable grassland area [5]. These noxious weed species are diverse, including multiple families such as Asteraceae, Fabaceae, Euphorbiaceae, Thymelaeaceae, and Ranunculaceae. Among them, dominant and common species include *Ageratina adenophora* (Spreng.) R.M.King & H.Rob., *Chromolaena odorata* (L.) R.M.King & H.Rob., *Bidens pilosa* L., *Euphorbia jolkinii* Boiss., and *Ligularia dictyoneura* (Franch.) Hand.–Mazz. [6,7,8]. Their harmful effects primarily include niche occupation and toxicity, with some species also exhibiting allelopathic effects. By inhibiting desirable forage growth, they form monodominant populations, leading to decreased grassland productivity and a sharp decline in biodiversity.

The distribution of noxious weeds in Yunnan exhibits distinct regional and altitudinal gradient characteristics: the southern thermo-therophytic shrub–grassland region is dominated by alien invasive species such as *Ageratina adenophora* and *Chromolaena odorata*, with high occurrence density and rapid spread; the central warm-temperate therophytic grassland region is negatively affected by *B. Pilosa* and ferns (*Pteridium* spp.); and the northern high-altitude mountain meadow region is primarily affected by native noxious weeds such as *E. jolkinii* and *L. dictyoneura*. Their invasion significantly alters soil nutrient cycling and microbial community structure, creating feedback effects that favor their own growth and further intensify population expansion [9]. In recent years, with climate warming and increased intensity of human activities, noxious weeds have shown a trend of continuous spread from high- to low-density areas, with particularly severe impacts in some localities [10]. For instance, in the thermo-therophytic shrub–grasslands of Tengchong City, the occurrence frequencies of *Ageratina adenophora* and *Bidens Pilosa* reach as high as 45.9% and 19.2%, respectively, forming absolute dominant populations assessed as high-risk [8]. In Yuxi City, the importance value of *Ageratina adenophora* is 44.48%, and its suitable area accounts for 66.71% of the city’s total land area, with *Bidens alba* (L.) DC., *Chromolaena odorata*, and ferns (*Pteridium* spp.) showing clear outward range expansion from high-density areas [7]. In Huize County, *Ageratina adenophora* causes severe damage below 1800 m altitude, ferns dominate in the 2000–2700 m zone, and *Sambucus adnata* Wall. ex DC. prevails above 3000 m, with the average yield of mixed noxious weeds reaching ~2143.857 kg/km^2^ while edible forage accounts for only 22.5% [11]. The harm caused by noxious weeds in these areas has seriously threatened grassland ecological security and livestock production. Comprehensively understanding the species composition, distribution patterns, occurrence characteristics, and ecological impacts of noxious weeds, in addition to assessing their range shifts, has become a critical scientific issue requiring urgent resolution for grassland conservation and restoration in Yunnan Province.

In this context, scientifically assessing the potential distribution and dispersal risks of noxious weeds requires the use of species distribution models (SDMs) to predict occurrence trends [12]. These models algorithmically correlate known occurrence points of target species with relevant environmental factors to infer species’ niche requirements and project the results onto specific spatiotemporal conditions, thereby predicting potential suitable habitats [13,14]. Commonly used SDMs include Maximum Entropy (MaxEnt), BIOCLIM, Ecological Niche Factor Analysis (ENFA), Classification and Regression Trees (CARTs), Generalized Linear Models (GLMs), Generalized Additive Models (GAMs), and Genetic Algorithm for Rule-set Production (GARP) [15,16,17,18]. The MaxEnt model, due to its high predictive accuracy, ease of operation, and strong adaptability to small sample sizes, performs excellently among these models and has become the most widely used tool for predicting species distributions [19].

For example, Yao et al. used MaxEnt on *Woonyoungia septentrionalis* and showed that temperature factors were the key variables that dominated its distribution [20]. Similarly, a study by Sun et al. on *Solanum aculeatissimum* in Southwest China found that human activity disturbance and isothermality were the dominant drivers [21]. Furthermore, a recent comprehensive review by López-Tirado and Gonzalez-Andujar (2023) synthesized 59 studies on weed distribution modeling published since 2017, revealing several key trends relevant to our work [22]. The authors found that MaxEnt is the most widely used algorithm (employed in 71.2% of reviewed studies), with AUC being the preferred validation metric—a methodological approach we adopted in this study. More importantly, their review highlighted that while Asteraceae and Poaceae together account for 45% of modeled weed species (with *Ageratina adenophora* being among the most frequently studied), most research has focused on global or continental scales, leaving regional-scale predictions for local management underdeveloped. They also underscored the need for high-resolution, species-specific predictions at the regional level that can directly inform differentiated management strategies for economically important weeds—a gap that the present study aims to address by focusing on Yunnan Province’s grassland ecosystems.

Despite the utility of previous SDM studies, critical gaps remain. Most rely on biased public databases, focus on single invasive species, and lack direct invasive–native comparisons under identical modeling conditions. Consequently, three key questions remain unanswered. First, do invasive and native Asteraceae weeds differ in their responses to key environmental drivers (climatic, edaphic, topographic, and anthropogenic), and, if so, what dominant factors shape their distributions? Second, under future climate scenarios, will invasive species exhibit net range expansion while native species undergo unidirectional habitat loss, or will their dynamics be more complex? Third, do these contrasting responses imply that invasive and native problematic weeds require fundamentally different management strategies? To answer these questions, we leverage high-resolution field survey data from the Yunnan Provincial Grassland Pest Survey. Using an optimized MaxEnt model with multi-source environmental variables, we compare two invasive species (*Chromolaena odorata*, *Ageratina adenophora*) and one native species (*Cirsium japonicum*). The findings are expected to provide a trait-based rationale for differentiating management strategies between expanding invasive weeds and a declining native medicinal species in Yunnan grasslands.

## 2. Results

### 2.1. Species Distribution and Comparative Bioecological Characteristics

**Figure 1:**
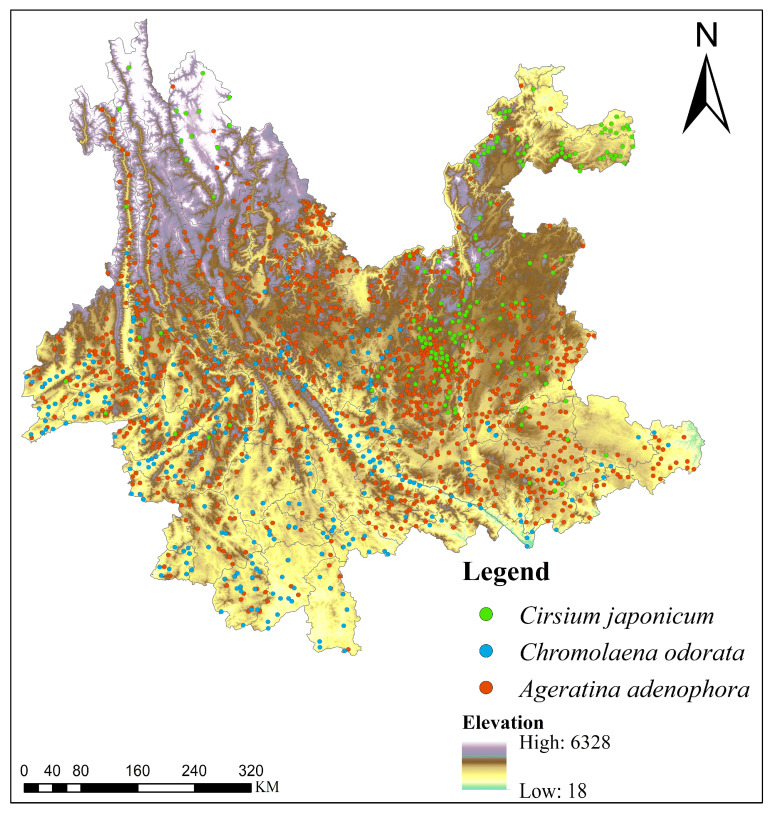
Distribution of survey occurrence points of three Asteraceae species (*Chromolaena odorata*, *Ageratina adenophora*, and *Cirsium japonicum*) in Yunnan Province.

Based on the field survey data from the Yunnan Provincial Grassland Pest Survey, the distribution patterns of the three Asteraceae species exhibit pronounced spatial differentiation (Figure 1). *Ageratina adenophora* is the most widely distributed and abundant species, with dense occurrence points covering most of Yunnan Province, particularly concentrated in the middle, southern, and eastern low- to mid-elevation regions. Its distribution markedly decreases in the northwestern high-altitude areas, though scattered occurrences remain. *Chromolaena odorata* shows a narrower distribution range, primarily confined to the southern and southwestern parts of Yunnan, with relatively scattered points and lower density than *Ageratina adenophora*. Elevation-wise, it mainly occurs in low-altitude river valleys and tropical/subtropical zones. In contrast, *Cirsium japonicum* displays clear regional aggregation, concentrating in the northeastern, eastern, and parts of northwestern Yunnan. Compared to the two invasive species, *Cirsium japonicum* tends to occupy higher elevations, with substantial occurrences in the northwestern high-altitude mountains. Overall, *Ageratina adenophora* is the dominant species with the broadest distribution and strongest adaptability, covering most low- to mid-elevation areas of the province; *Chromolaena odorata* is largely restricted to low-altitude tropical/subtropical regions in the south and southwest; and *Cirsium japonicum* exhibits a distinct preference for higher elevations in the northeast, east, and northwest.

The contrasting distribution patterns are further supported by the bioecological characteristics of the three species, which were obtained from field surveys and the literature (Table 1). *Ageratina adenophora* displayed the highest importance value (31.5), density (18.6 plants/m^2^), and cover (32.5%), confirming its status as the absolute dominant species. Its broad ecological niche, combined with sexual and vegetative reproduction (30,000–150,000 seeds per plant), strong allelopathy, and tolerance to diverse soils and moderate disturbance, underpins its widespread occurrence. *Chromolaena odorata*, though ranking second in importance value (24.2) and cover (26.3%), exhibited the greatest height (82.1 cm) and a preference for sandy, infertile soils in warm, humid lowlands. Its high seed output, wind dispersal, and fire resistance explain its concentration in southern low-altitude areas. By contrast, the native *Cirsium japonicum* had the lowest importance value (8.2), density (2.5 plants/m^2^), and cover (7.2%), with a narrow niche favoring cool-temperate (10–18 °C), moist, clay-rich, fertile soils. Its weak allelopathy, limited dispersal capacity, and high temperature sensitivity restrict it to higher elevations in northeastern and northwestern Yunnan, where it typically occurs as a subordinate species. The higher seed output, wind dispersal, and allelopathy of invasive species explain their wider distribution, while the limited dispersal and narrow thermal optimum of *Cirsium japonicum* explain its restricted range.

### 2.2. Model Accuracy Evaluation

**Figure 2:**
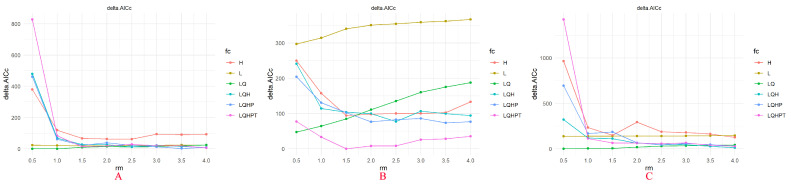
AICc model selection of MaxEnt models for *Chromolaena odorata* (**A**), *Ageratina adenophora* (**B**) and *Cirsium japonicum* (**C**).

**Figure 3:**
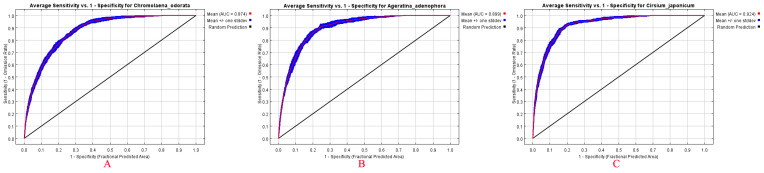
ROC curve validation of MaxEnt models for *Chromolaena odorata* (**A**), *Ageratina adenophora* (**B**) and *Cirsium japonicum* (**C**).

The AICc-based model selection results indicated (Figure 2) that the MaxEnt models for *Chromolaena odorata*, *Ageratina adenophora*, and *Cirsium japonicum* all identified optimal feature class (FC) and regularization multiplier (RM) combinations. The optimal model for *Chromolaena odorata* and *Cirsium japonicum* was LQ (linear + quadratic features), while for *Ageratina adenophora*, it was LQHPT (linear + quadratic + hinge + product + threshold features) with delta. AICc values approached the minimum (0). ROC curve validation demonstrated (Figure 3) that the predictive performance of the models for all three species was significantly better than random (AUC = 0.5). Regarding mean AUC values, *Cirsium japonicum* had the highest (0.924 ± 0.006), *Chromolaena odorata* had the lowest (0.874 ± 0.007), and *Ageratina adenophora* had an intermediate value (0.899 ± 0.009). Specificity was defined as the proportional predicted area, effectively excluding interference from the true negative rate and further confirming model reliability. The differences in AUC values among the three species (*Cirsium japonicum* > *Ageratina adenophora* > *Chromolaena odorata*) were consistent with their ecological characteristics; in particular, the native species *Cirsium japonicum* showed more precise adaptability to the Yunnan Plateau habitat, while the predictive accuracy for the invasive species was relatively lower, reflecting their differential sensitivity to environmental thresholds.

### 2.3. Key Influencing Factors

**Figure 4:**
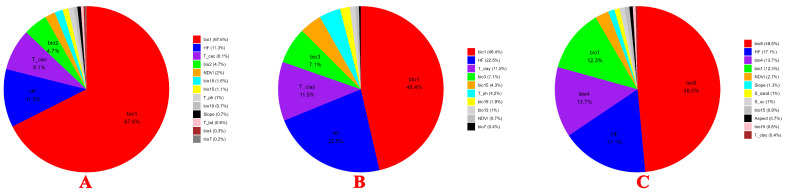
Percent contribution of key environmental variables to MaxEnt models for *Chromolaena odorata* (**A**), *Ageratina adenophora* (**B**), and *Cirsium japonicum* (**C**).

Analysis of the environmental variable contribution rates for *Chromolaena odorata* (A), *Ageratina adenophora* (B), and *Cirsium japonicum* (C) revealed the main drivers of their distributions. The results showed (Figure 4) that mean annual temperature (BIO1) was the core climatic factor for all three species. *Chromolaena odorata* was most sensitive to mean annual temperature, with a contribution rate reaching 67.6%, significantly higher than those of *Ageratina adenophora* (12.3%) and *Cirsium japonicum* (46.4%). The distribution of *Ageratina adenophora* was primarily driven by the mean temperature of the driest quarter (BIO9, 48.5%), indicating its strong adaptability to dry season temperature conditions. Human Footprint (HF) played a significant role in the distribution of all three species, with contribution rates of 11.3% for *Chromolaena odorata*, 17.1% for *Ageratina adenophora*, and 22.5% for *Cirsium japonicum*, suggesting a more pronounced influence of human activities on *Ageratina adenophora* and *Cirsium japonicum*. Regarding soil factors, *Chromolaena odorata* was constrained by topsoil cation exchange capacity (T_cec, 8.1%), while *Ageratina adenophora* and *Cirsium japonicum* showed strong responses to soil clay content (T_clay, both at 11.5%). Furthermore, *Ageratina adenophora* was sensitive to the standard deviation of temperature seasonality (BIO4, 13.7%), and *Cirsium japonicum* was influenced by isothermality (BIO3, 7.1%), reflecting their differential adaptation to climatic seasonal stability. In summary, while mean annual temperature was the core driver for all three species, *Ageratina adenophora* relied more on dry season temperature and temperature seasonality, *Cirsium japonicum* and *Ageratina adenophora* were more significantly affected by human activity and soil clay content, and *Chromolaena odorata* was more sensitive to soil cation exchange capacity.

**Figure 5:**
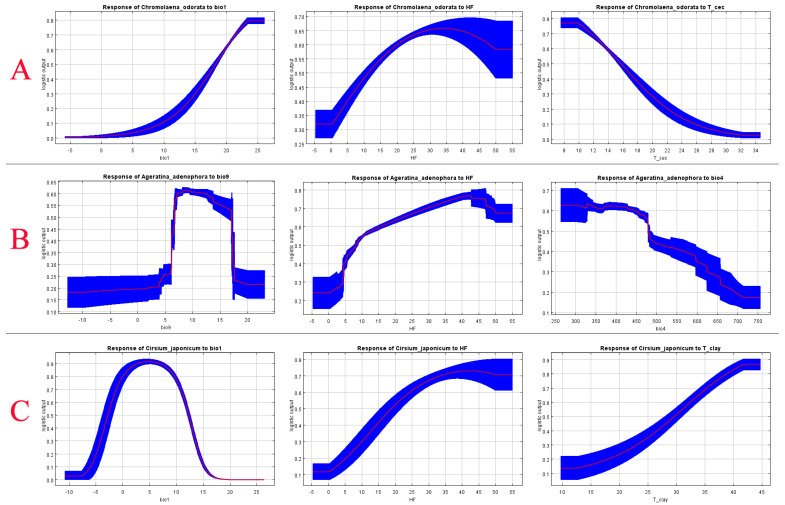
Response curves of the top three dominant factors for *Chromolaena odorata* (**A**), *Ageratina adenophora* (**B**), and *Cirsium japonicum* (**C**).

To further explore the influence of the core drivers, the response curves for the top three environmental factors for each species were analyzed. The results indicated the following (Figure 5):

*Chromolaena odorata* showed a sustained positive response to mean annual temperature (BIO1) (suitability continuously increased from near 0 to 0.8 as BIO1 rose from −5 °C to 25 °C), a unimodal response to Human Footprint (HF) (suitability peaked at 0.65–0.7 when the HF index was 20–40), and a negative response to soil cation exchange capacity (T_cec) (suitability rapidly decreased from 0.8 to near 0 as T_cec increased from 8 to 34 cmol/kg). This indicates a preference for habitats with warm climates, moderate disturbance, and low cation exchange capacity soils (e.g., sandy/infertile soils).

*Ageratina adenophora* exhibited a unimodal response to mean temperature of the driest quarter (BIO9) (suitability remained around 0.6 when BIO9 was 10–15 °C, declining significantly outside this range), a unimodal response to Human Footprint (HF) (suitability peaked at 0.8 when the HF index was 40–50), and a negative response to the standard deviation of temperature seasonality (BIO4) (suitability decreased from 0.7 to 0.2 as BIO4 increased from 250 to 750). This reflects its adaptation to habitats with moderate dry season temperatures, moderate-to-high disturbance, and low climatic seasonality.

*Cirsium japonicum* showed a strictly unimodal response to mean annual temperature (BIO1) (suitability peaked above 0.7 when BIO1 was 0–10 °C, decreasing rapidly outside this range, indicating sensitivity to high temperatures), a unimodal response to Human Footprint (HF) (suitability ~0.8 when the HF index was 40–50), and a positive response to soil clay content (T_clay) (suitability increased from 0.2 to 0.9 as T_clay increased from 10% to 45%). This indicates a preference for cool climates, moderate-to-high disturbance, and clay-rich soils.

### 2.4. Suitable Habitats of the Three Species Under the Current Climate

#### 2.4.1. *Chromolaena odorata*

**Figure 6:**
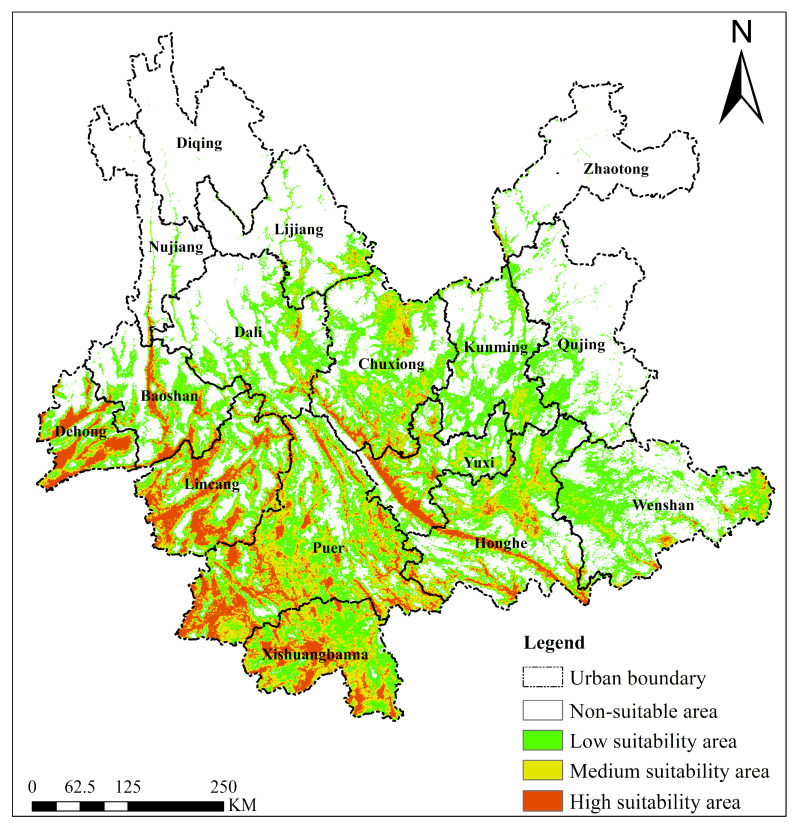
Suitable habitat distribution of *Chromolaena odorata* in Yunnan Province under the current climate scenario.

The predicted current suitable habitat for *Chromolaena odorata* exhibited significant spatial differentiation (Figure 6). Highly suitable areas were mainly concentrated in western Yunnan (Dehong, Baoshan, Lincang), southern Yunnan (Puer, Xishuangbanna), and parts of Honghe Prefecture. These regions are characterized by tropical and south subtropical monsoon climates with high annual mean temperatures, abundant precipitation, and altitudes mostly ranging from 500 to 1500 m (e.g., river valleys and basins). Influenced by north–south-oriented mountain ranges such as the Gaoligong Mountains, the low-altitude areas receive abundant orographic precipitation, resulting in superior hydrothermal conditions that provide highly suitable habitats for *Chromolaena odorata*, covering an area of approximately ~2.78 × 10^4^ km^2^. Medium-suitability areas were scattered around the highly suitable zones and in central Yunnan (Chuxiong), where hydrothermal conditions are slightly weaker than in the south but still offer considerable suitability, covering approximately ~4.45 × 10^4^ km^2^. Low-suitability areas were widely distributed across regions outside northwestern and northeastern Yunnan, covering approximately ~9.05 × 10^4^ km^2^. Unsuitable areas were located in high-altitude or arid regions, such as Diqing and northern Zhaotong, characterized by cold climates (low annual mean temperature) or severe aridity, with altitudes exceeding 3000 m or insufficient hydrothermal conditions unable to support *Chromolaena odorata* growth, covering ~1.76 × 10^5^ km^2^. Overall, the suitability for *Chromolaena odorata* is closely linked to hydrothermal conditions coupled with latitude, altitude, and topography. Tropical and south subtropical low-altitude mountainous areas constitute its core suitable zones, while topographically complex areas (e.g., eastern foothills of the Gaoligong Mountains) harbor local high-suitability patches due to significant vertical differentiation.

#### 2.4.2. *Ageratina adenophora*

**Figure 7:**
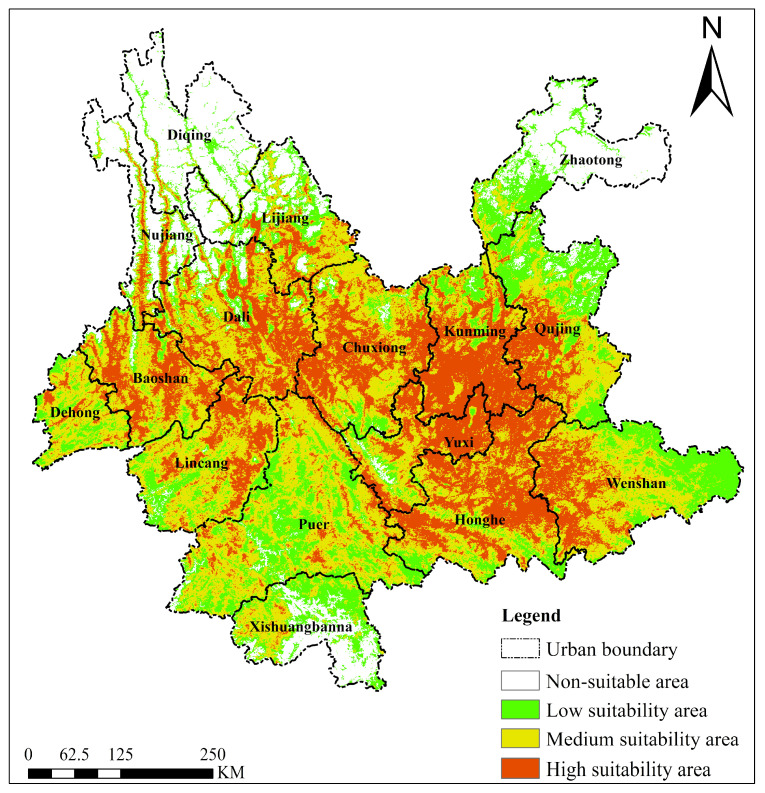
Suitable habitat distribution of *Ageratina adenophora* in Yunnan Province under the current climate scenario.

The predicted current suitable habitat for *Ageratina adenophora* also exhibited significant spatial differentiation (Figure 7). Highly suitable areas were distributed across multiple locations, primarily concentrated in central Yunnan (e.g., Chuxiong, Yuxi, southern Kunming). This region is characterized by a warm and humid climate (annual mean temperature 18–22 °C; annual precipitation 1200–1500 mm) and flat terrain conducive to habitat expansion, covering ~9.12 × 10^4^ km^2^ (approximately 26.9% of the province’s total area). Medium-suitability areas extensively covered the east (Wenshan, Honghe), south (northern Puer, Xishuangbanna), and parts of the west (Baoshan, Dehong), where suitability slightly decreased due to increased elevation (1500–2500 m) or precipitation fluctuations, covering ~12.19 × 10^4^ km^2^ (approximately 36.0%). Low-suitability areas were widely distributed in southern Yunnan (Pu’er, Xishuangbanna) and parts of eastern Yunnan (Wenshan and Qujing), covering ~6.80 × 10^4^ km^2^ (approximately 20.1%). Unsuitable areas were mainly located in the high-altitude cold regions of the northwest (northern Diqing, northern Nujiang), with annual mean temperatures below 10 °C and short growing seasons, covering ~5.75 × 10^4^ km^2^ (approximately 17.0%). Overall, the suitability for *Ageratina adenophora* is tightly coupled with hydrothermal conditions and altitudinal gradients. The medium-suitability area is the largest (accounting for 36.0%), indicating a widespread invasion risk for *Ageratina adenophora* in Yunnan Province. Its core distribution is highly dependent on warm, humid low-altitude areas in the central and southern parts, while topographically complex areas (e.g., around the Ailao and Wuliang Mountains) harbor local high-suitability patches.

#### 2.4.3. *Cirsium japonicum*

**Figure 8:**
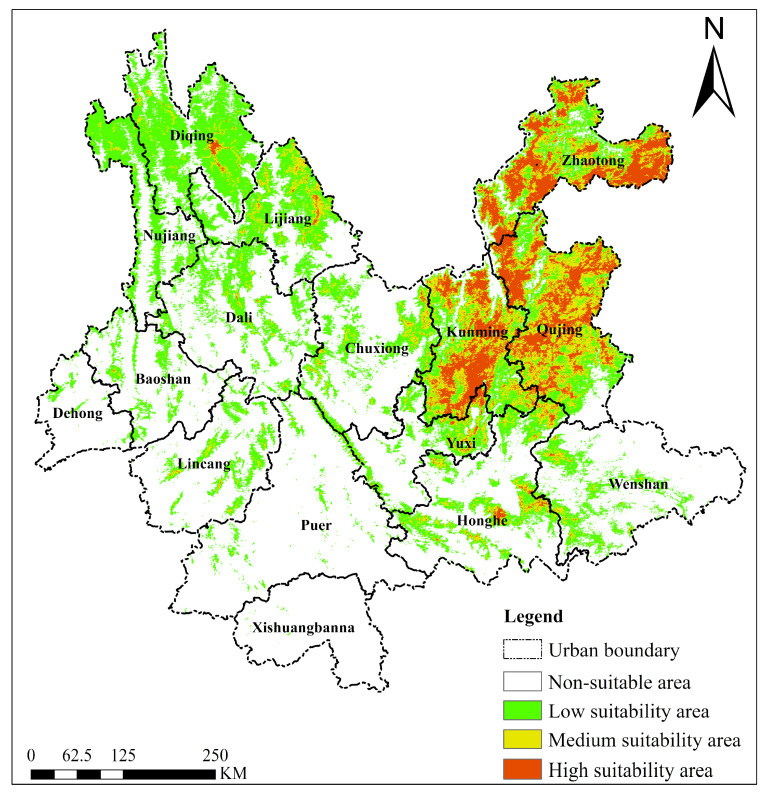
Suitable habitat distribution of *Cirsium japonicum* in Yunnan Province under the current climate scenario.

As a native species of Yunnan Province, the predicted current suitable habitat for *Cirsium japonicum* (Figure 8) exhibited distinct climate–topography coupling characteristics. Highly suitable areas (approximately 2.29 × 10^4^ km^2^) were concentrated in central and northeastern Yunnan (Qujing, Kunming, Zhaotong, etc.), with an average elevation of approximately 1500–2000 m. These areas are characterized by a plateau monsoon climate (annual mean temperature 10–18 °C; annual precipitation 800–1200 mm), flat terrain, and fertile soils, providing ideal micro-environments for the native growth of *Cirsium japonicum*. Medium-suitability areas (approximately 2.92 × 10^4^ km^2^) were distributed in small patches in central and eastern Yunnan, where suitability slightly decreased due to precipitation fluctuations and topographic slope. Low-suitability areas (approximately 8.09 × 10^4^ km^2^) were mainly distributed in western Yunnan and the northwest (Diqing, Lijiang, Nujiang), where complex terrain and significant altitudinal differences greatly influence suitability. Unsuitable areas were mainly concentrated in southern Yunnan (e.g., Puer, Xishuangbanna), constrained by high altitudes (2000–3000 m) in mountain ranges, such as the Gaoligong Mountains, or by tropical humid and hot environments. Overall, the suitability of *Cirsium japonicum* shows a strong positive correlation with the moderately temperate and humid plateau environment. Its distribution pattern as a native species is highly dependent on hydrothermal gradients and topographic barriers (e.g., Gaoligong Mountains) on the Yunnan–Guizhou Plateau.

#### 2.4.4. Overlay Analysis of Suitable Habitats

**Figure 9:**
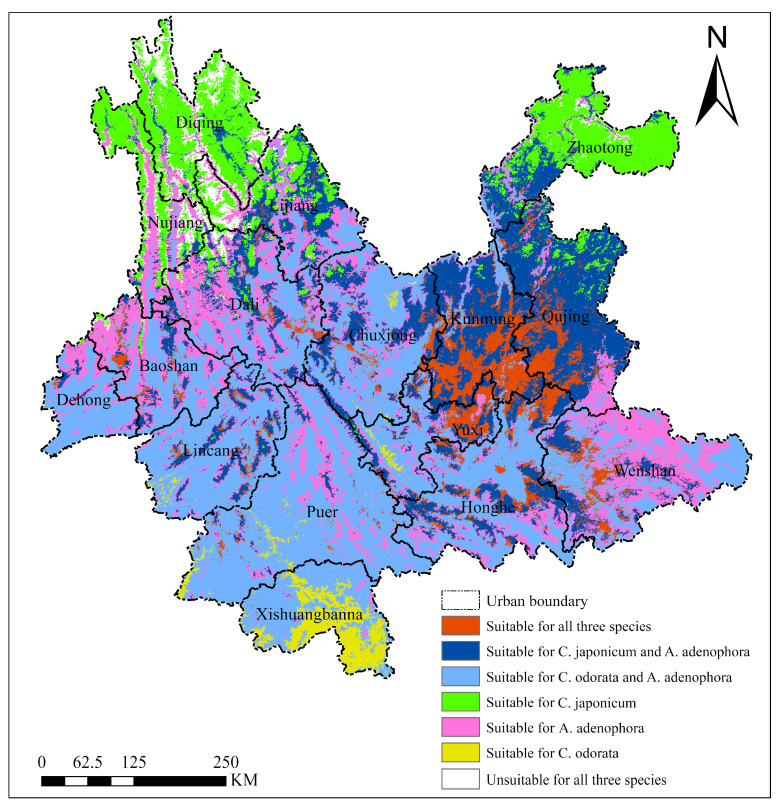
Overlay analysis of suitable habitats for *Ageratina adenophora* (*A. adenophora*), *Chromolaena odorata* (*C. odorata*), and *Cirsium japonicum* (*C. japonicum*) in Yunnan Province.

Overlay analysis of the current potential suitable habitat maps (Figure 9) revealed significant geographic differentiation and differences in environmental adaptability among the two invasive species (*Ageratina adenophora* and *Chromolaena odorata*) and the native species (*Cirsium japonicum*). The combined suitable area for *Ageratina adenophora* and *Chromolaena odorata* was the largest (~13.23 × 10^4^ km^2^), covering low-latitude tropical to subtropical regions such as central and southern Yunnan. The exclusive suitable area for *Cirsium japonicum* (~4.05 × 10^4^ km^2^), a native noxious weed, was concentrated in northern high-altitude temperate regions such as Diqing and Lijiang, reflecting its preference for specific environments, but the area was relatively small. The overlapping area for all three species (~2.25 × 10^4^ km^2^) was mainly distributed around central cities such as Kunming and Qujing. The unsuitable area for all three species was only ~0.90 × 10^4^ km^2^, indicating that the vast majority of areas in Yunnan Province are suitable for at least one of the invasive species, posing an urgent challenge for regional biological invasion control and native plant conservation.

### 2.5. Contraction and Expansion Dynamics of Suitable Habitats for the Three Species Under Future Climate Scenarios

#### 2.5.1. Dynamics of *Chromolaena odorata* Under Three SSP Scenarios for the 2030s and 2050s

**Figure 10:**
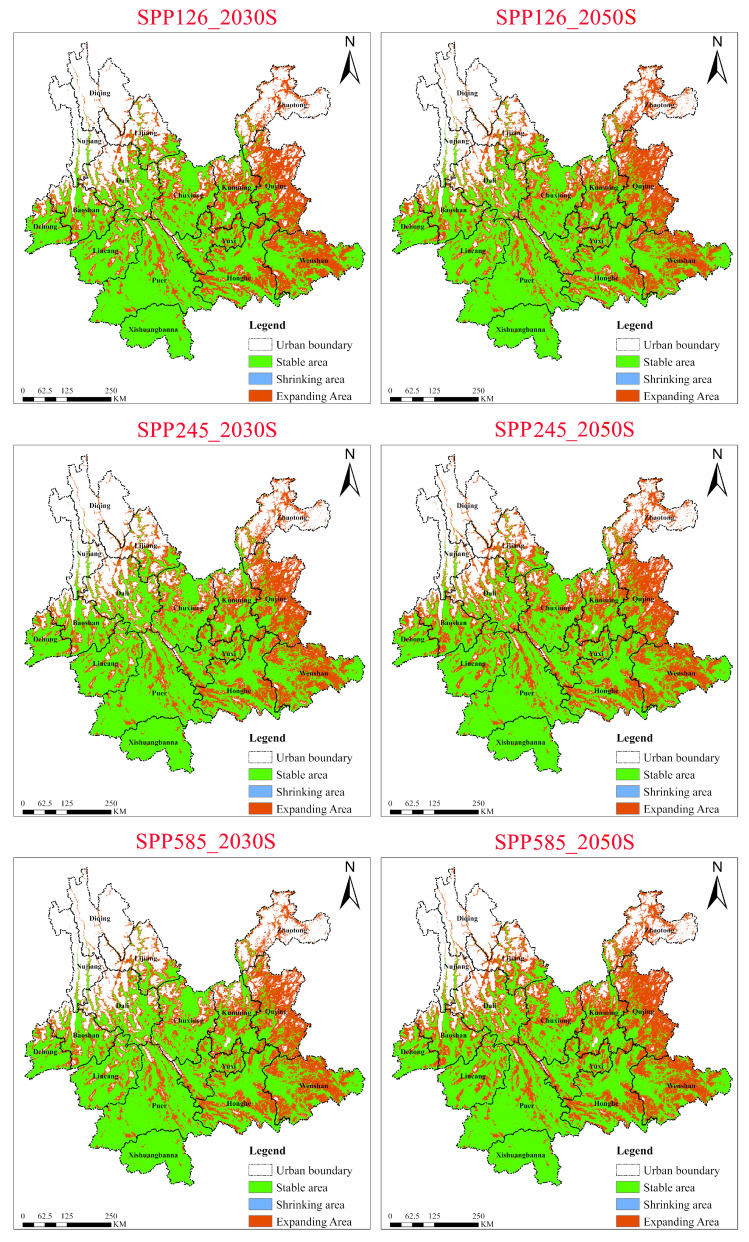
Dynamics of suitable habitat contraction and expansion areas for *Chromolaena odorata* in Yunnan Province under different climate scenarios (2030s, 2050s).

Under the low-emission scenario (SSP1-2.6), the suitable habitat of *Chromolaena odorata* exhibited stability with moderate expansion (Figure 10). The stable area remained dominant, maintaining ~16.28 × 10^4^ km^2^ in the 2030s and ~16.27 × 10^4^ km^2^ in the 2050s, indicating high resilience of the species’ niche in its current core distribution areas. Notably, the contraction area was extremely small and decreased significantly over time, dropping sharply from ~0.0024 × 10^4^ km^2^ in the 2030s to 0.00071 × 10^4^ km^2^ in the 2050s. Concurrently, the expansion area increased from ~9.09 × 10^4^ km^2^ to ~9.92 × 10^4^ km^2^. Spatially, by the 2050s, stable areas broadly covered southwestern and central Yunnan, while expansion areas were mainly distributed in eastern Yunnan (Kunming, Qujing), northeastern Yunnan (Zhaotong), and southeastern Yunnan (Wenshan), with contraction areas nearly disappearing. This indicates that even under a relatively minor climate warming scenario, *Chromolaena odorata* still shows a net increase, with some previously unsuitable habitats gradually becoming suitable.

Under the medium-emission scenario (SSP2-4.5), *Chromolaena odorata* displayed a more pronounced expansion trend, with further enhanced environmental adaptability. Between the 2030s and 2050s, the stable area remained constant while the contraction area completely disappeared, implying no significant risk of habitat loss under this scenario. In contrast, the expansion area grew from ~9.51 × 10^4^ km^2^ to ~10.37 × 10^4^ km^2^, a greater increase than under SSP1-2.6. Spatially, over time (from the 2030s to the 2050s), previously scattered expansion patches showed an increased trend of coalescence in eastern Yunnan (Qujing), northeastern Yunnan (Zhaotong), and southeastern Yunnan (Wenshan), indicating that moderate climate change will promote the rapid infiltration of *Chromolaena odorata* into higher latitudes or altitudes.

Under the high-emission scenario (SSP5-8.5), the driving effect of climate warming on the distribution of *Chromolaena odorata* was most intense, manifested as the largest-scale habitat expansion. In this scenario, the contraction area also completely disappeared by the 2050s and the expansion area reached its maximum among the three scenarios, increasing from ~9.24 × 10^4^ km^2^ in the 2030s to ~10.68 × 10^4^ km^2^ in the 2050s. Compared to the first two scenarios, the increment in expansion under SSP5-8.5 was the most significant, suggesting that extreme climate conditions may break the original temperature constraints, greatly enabling *Chromolaena odorata* range expansion. Spatially, the coverage density of expansion areas increased markedly across the entire province, especially in the northeastern and eastern marginal zones. In summary, regardless of the emission scenario, *Chromolaena odorata* exhibited a stable and continuously expanding trend. As radiative forcing increased, its contraction risk approached zero and its expansion potential was progressively amplified, indicating that future climate change will favor the further establishment and spread of this invasive species in Yunnan.

#### 2.5.2. Dynamics of *Ageratina adenophora* Under Three SSP Scenarios for the 2030s and 2050s

**Figure 11:**
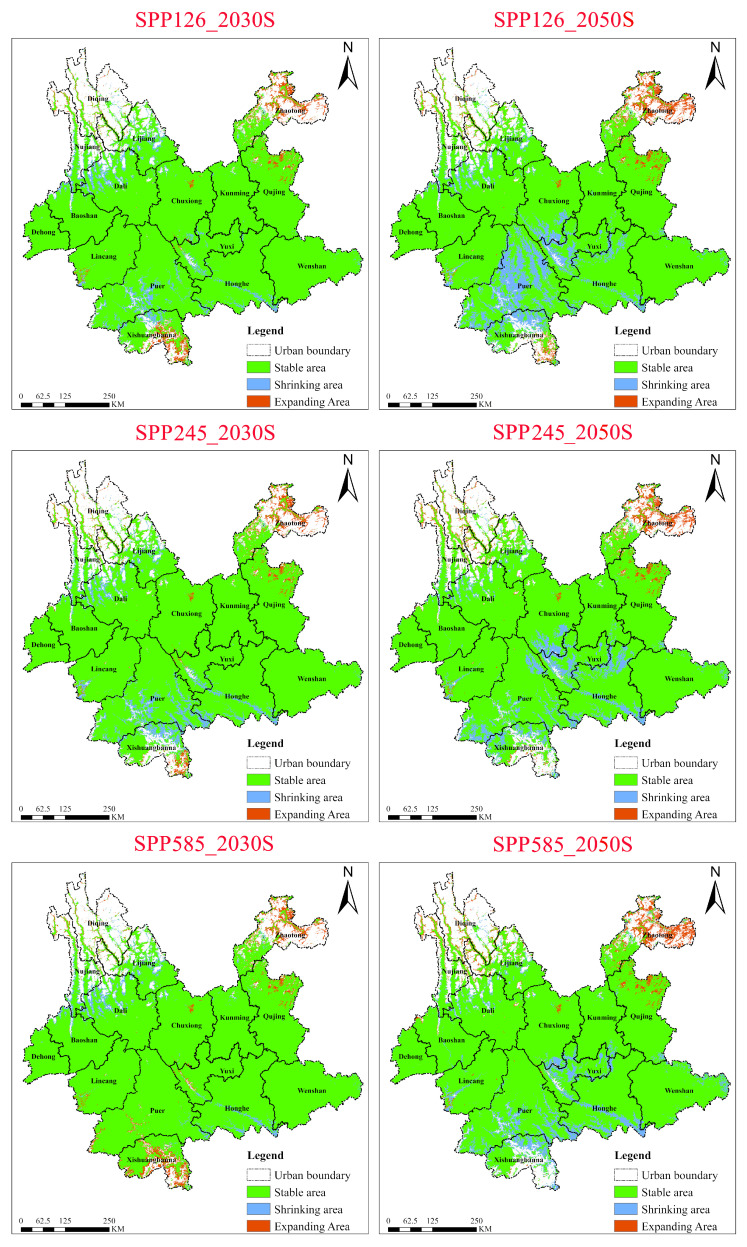
Dynamics of suitable habitat contraction and expansion areas for *Ageratina adenophora* in Yunnan Province under different climate scenarios (2030s, 2050s).

Under the low-emission scenario (SSP1-2.6), the suitable habitat of *Ageratina adenophora* showed a slight contraction pattern (Figure 11). From the 2030s to the 2050s, the stable area decreased from 26.56 × 10^4^ km^2^ to 24.59 × 10^4^ km^2^, while the contraction area increased from ~1.54 × 10^4^ km^2^ to ~3.51 × 10^4^ km^2^ (an increase of 128%). The expansion area increased only slightly. This indicates that, while climate warming did not significantly expand its overall range, it accelerated habitat degradation in the low-altitude, humid, and hot areas of the south (e.g., Xishuangbanna, Puer). Spatially, by the 2050s, contraction patches expanded contiguously in the river valley areas of southwestern Yunnan (Lincang, Puer, Xishuangbanna), while expansion was limited to high-altitude marginal zones such as northeastern Yunnan (Zhaotong, northern Qujing).

Under the medium-emission scenario (SSP2-4.5), the dynamics of *Ageratina adenophora*’s suitable habitat were similar to those under SSP1-2.6, but the contraction rate was slightly lower. From the 2030s to the 2050s, the stable area decreased from ~26.35 × 10^4^ km^2^ to ~25.56 × 10^4^ km^2^ (a reduction of 3.0%), the contraction area increased from ~1.76 × 10^4^ km^2^ to ~3.51 × 10^4^ km^2^ (an increase of 100%), and the expansion area increased slightly. This suggests that under moderate warming, the species’ response to habitat degradation tends to stabilize. In the 2050s, contraction areas were concentrated in the subtropical river valleys of central and southwestern Yunnan (Yuxi, Puer, etc.). Notably, small local patches of expansion formed in northwestern Yunnan (Lijiang, Diqing), highlighting its adaptive migration capacity along altitudinal gradients.

Under the high-emission scenario (SSP5-8.5), the suitable habitat dynamics of *Ageratina adenophora* presented a complex response characterized by initial stability followed by contraction, coupled with local expansion. The stable area was largest in the 2030s (~27.12 × 10^4^ km^2^), and the contraction area was the smallest (~0.98 × 10^4^ km^2^). However, by the 2050s, the stable area sharply decreased to ~25.68 × 10^4^ km^2^ (a reduction of 5.3%), the contraction area increased to ~2.42 × 10^4^ km^2^ (an increase of 146%), and the expansion area concurrently increased to ~1.16 × 10^4^ km^2^ (an increase of 5.5%). This indicates that, under extreme climate conditions, adaptive expansion of the species in high-altitude areas (e.g., northwestern Yunnan) partially offsets habitat loss at lower altitudes. By the 2050s, contraction areas were concentrated in the southern tropical zone (Xishuangbanna), while expansion areas developed contiguously in parts of northwestern Yunnan (Diqing, Lijiang) and northeastern Yunnan (Zhaotong). This suggests that rising temperatures may enable it to breach high-altitude barriers. However, suitability in the southern humid and hot areas remains significantly weakened, forming a pattern of southern contraction versus northwestern and northeastern expansion.

#### 2.5.3. Dynamics of *Cirsium japonicum* Under Three SSP Scenarios for the 2030s and 2050s

**Figure 12:**
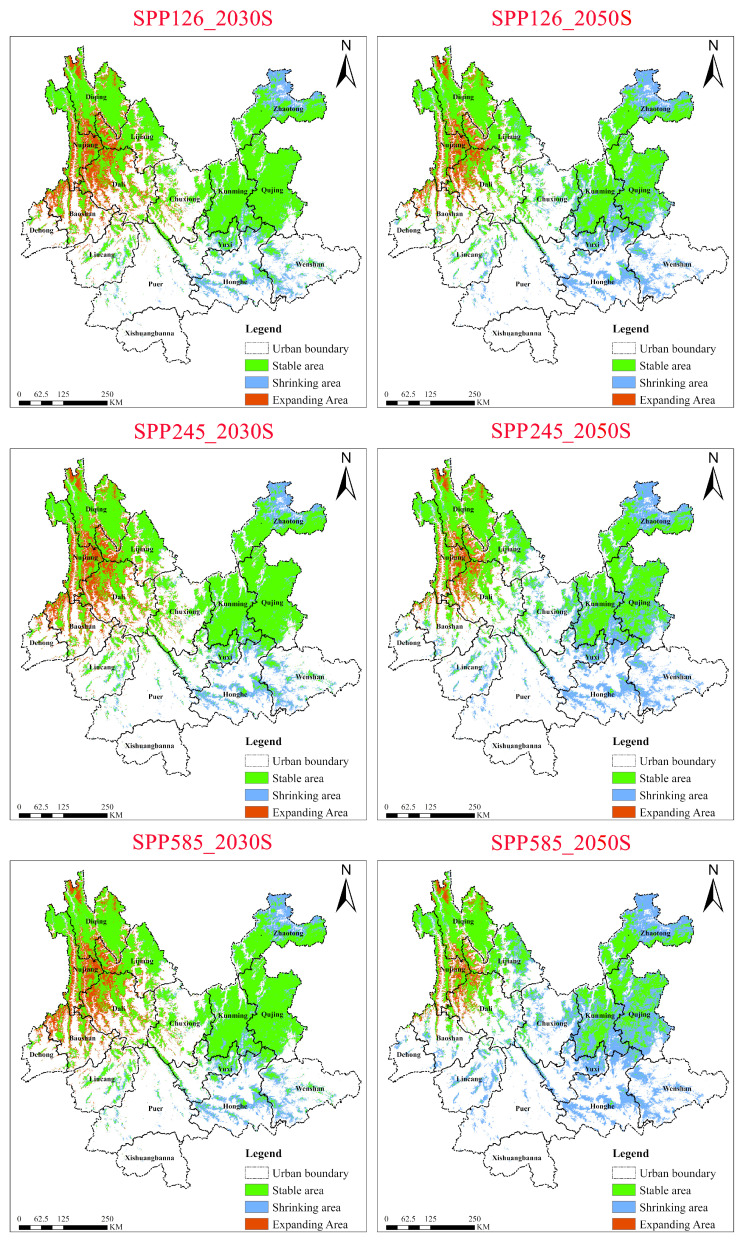
Dynamics of suitable habitat contraction and expansion areas for *Cirsium japonicum* in Yunnan Province under different climate scenarios (2030s, 2050s).

Under the low-emission scenario (SSP1-2.6), *Cirsium japonicum* exhibited a pattern of continuous habitat contraction (Figure 12). From the 2030s to the 2050s, the stable area decreased from ~10.85 × 10^4^ km^2^ to ~8.97 × 10^4^ km^2^ (a reduction of 17.3%), the contraction area increased from ~2.45 × 10^4^ km^2^ to ~4.33 × 10^4^ km^2^ (an increase of 76.7%), and the expansion area shrank from ~2.67 × 10^4^ km^2^ to ~1.69 × 10^4^ km^2^ (a reduction of 36.5%). This indicates that even modest climate warming significantly weakened its core suitable areas (e.g., the central Yunnan Plateau). Spatially, contraction patches expanded contiguously in southeastern Yunnan (Honghe, Wenshan) while expansion was limited to parts of northwestern Yunnan (Dali, Baoshan, Nujiang), reflecting its sensitivity to temperature thresholds—habitat degradation is triggered even under low-intensity warming, but its migration capacity is limited.

Under the medium-emission scenario (SSP2-4.5), the rate of habitat degradation for *Cirsium japonicum* accelerated significantly. From the 2030s to the 2050s, the stable area decreased from ~10.75 × 10^4^ km^2^ to ~8.17 × 10^4^ km^2^ (a reduction of 24.0%), the contraction area expanded from ~2.55 × 10^4^ km^2^ to ~5.13 × 10^4^ km^2^ (an increase of 101.0%), and the expansion area further shrank from ~2.51 × 10^4^ km^2^ to ~1.30 × 10^4^ km^2^ (a reduction of 48.4%). By the 2050s, contraction areas formed a continuous patch in central-southern Yunnan (Yuxi, Honghe) while expansion areas persisted only on high-altitude margins (e.g., parts of Nujiang, Diqing). This indicates that moderate warming caused the species to lose its ability to maintain core habitats, and its adaptive migration to higher altitudes was insufficient, leading to a continuous contraction of its distribution range.

Under the high-emission scenario (SSP5-8.5), *Cirsium japonicum* faced severe habitat degradation. The stable area sharply decreased to ~6.67 × 10^4^ km^2^ (from 10.66 × 10^4^ km^2^ in the 2030s, a reduction of 37.4%), the contraction area surged to ~6.63 × 10^4^ km^2^ (from ~2.64 × 10^4^ km^2^ in the 2030s, an increase of 151.0%), and the expansion area shrank to ~1.02 × 10^4^ km^2^ (a reduction of 58.4%). By the 2050s, the contraction area (~6.63 × 10^4^ km^2^) and the stable area (~6.67 × 10^4^ km^2^) were nearly equal, indicating that over 50% of the original suitable habitat may lose suitability. Spatially, contraction and unsuitable areas cover the main parts of southern and southwestern Yunnan, with only scattered stable patches of expansion remaining in northwestern Yunnan (Diqing, Nujiang, Dali).

In contrast to the invasive species *Chromolaena odorata* (expansion-dominated across all scenarios, with contraction risk approaching zero and large stable areas) and *Ageratina adenophora* (southern contraction compensated by northern expansion, also with large stable areas), the native species *Cirsium japonicum* exhibited unidirectional habitat degradation under all three scenarios, with the intensity of degradation intensifying as emission levels increased.

### 2.6. Average Center-Point Shift

**Figure 13:**
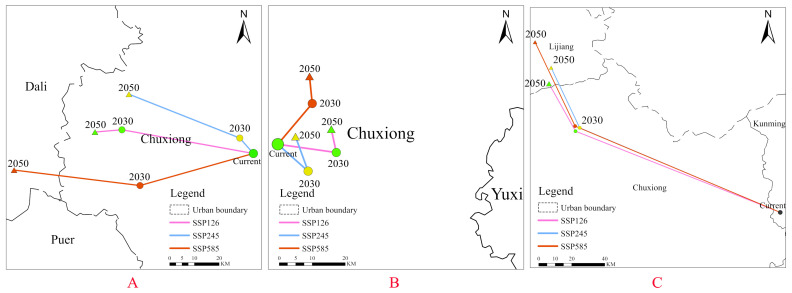
Average center-point shift trajectories of *Cirsium japonicum* (**A**), *Chromolaena odorata* (**B**), and *Ageratina adenophora* (**C**) from the 2030s to the 2050s.

Analysis of distribution centers indicated (Figure 13) that the centroid shift for the native species *Cirsium japonicum* was characterized by significant cross-regional migration, with its shift trajectory spanning three prefecture-level cities (Lijiang, Dali, Chuxiong), and the shift distance was markedly longer than that of the other two species. In contrast, the centroid shift ranges of the invasive species *Chromolaena odorata* and *Ageratina adenophora* were relatively limited: *Chromolaena odorata* spanned two cities (Kunming, Yuxi), while *Ageratina adenophora* exhibited only a minor shift within Chuxiong City. However, considering the contraction and expansion dynamic analysis, the invasive species still have the potential for further change (e.g., *Ageratina adenophora* potentially breaching high-altitude barriers under the high-emission scenario).

## 3. Discussion

### 3.1. Model Performance and Current Distribution Patterns

This study presents significant advantages in terms of the used data sources. Compared with previous species distribution modeling studies that often relied on online databases and literature records, we obtained primary data from the Yunnan Provincial Grassland Pest Survey, representing high-precision distribution records of noxious Asteraceae weeds. This data foundation offers higher spatial coverage and sampling consistency, effectively reducing model uncertainty caused by data bias and providing a reliable basis for accurate predictions. In this study, following a rigorous optimization process [23], the MaxEnt models for *Chromolaena odorata*, *Ageratina adenophora*, and *Cirsium japonicum* achieved AUC values of 0.874, 0.899, and 0.924, respectively, all meeting the criteria for “good” to “excellent” predictive accuracy [24]. This demonstrates the models’ high capability and reliability for predicting the potential distribution of these three noxious Asteraceae species in Yunnan Province.

Under the current climate scenario, the highly suitable areas for the two invasive species, *Chromolaena odorata* and *Ageratina adenophora*, were concentrated in the tropical river valleys of southwestern Yunnan (Dehong, Lincang, Xishuangbanna) and the plateau basin areas of central Yunnan (Chuxiong, Yuxi, southern Kunming), respectively. These findings are largely consistent with previous studies on their distribution ranges in southwestern China [25,26]. The highly suitable area for *Chromolaena odorata* is relatively small and highly dependent on low-altitude humid and hot micro-environments, consistent with its demanding hydrothermal requirements. *Ageratina adenophora* exhibits the most extensive suitable range, covering most of central Yunnan, reflecting its broad ecological amplitude and strong adaptability to various habitats. The highly suitable area for the native species *Cirsium japonicum* is concentrated in eastern (Qujing, Kunming) and northeastern (Zhaotong) Yunnan, displaying distinct climate–topography coupling characteristics, with its suitable areas restricted by mountain ranges such as the Gaoligong and Ailao Mountains to the core zone of the Yunnan–Guizhou Plateau [27]. Overlay analysis of suitable habitats further revealed complex spatial interactions among species: the combined suitable area for *Ageratina adenophora* and *Chromolaena odorata* extensively covers southwestern and southern Yunnan, indicating a potential risk of synergistic invasion. The overlapping area for all three species is concentrated around central cities such as Kunming and Qujing, suggesting possible niche competition and interactions between invasive and native noxious species in these regions [28]. The above distribution patterns are highly consistent with field-measured biological traits of the three species. *Ageratina adenophora* exhibited the highest importance value, density, and cover; coupled with its high seed output and strong allelopathy, this led to its broad suitable area [29]. *Chromolaena odorata* relies on its height advantage to occupy vertical space and tolerates drought and fire, allowing it to concentrate in low-altitude river valleys of southwestern Yunnan. In contrast, the native *Cirsium japonicum* had low importance value, density, and cover; it reproduces primarily by seeds and is sensitive to high temperatures, restricting its suitable area to the cool-temperate zones of central and northeastern Yunnan. High seed output, wind dispersal, and allelopathy of the invasive species match their wide suitable areas, whereas the low seed output, lack of allelopathy, and high temperature sensitivity of *Cirsium japonicum* match its narrow range.

### 3.2. Dominant Driving Factors and Future Responses

The contribution analysis distinguishes environmental from anthropogenic drivers. Among environmental factors, temperature regimes played the primary role. Mean annual temperature dominated for *Chromolaena odorata* (67.6% contribution) and *Cirsium japonicum* (46.4%), while dry season temperature was most important for *Ageratina adenophora* (48.5%). These climatic variables define the broad potential ranges of each species [30]. The sustained positive response of *Chromolaena odorata* to rising temperatures suggests that this tropical invasive species has not yet reached its thermal ceiling in Yunnan; further warming will likely expand its suitable habitat northward and eastward. In contrast, the strictly unimodal response of the native *Cirsium japonicum* (optimum 0–10 °C) indicates a narrow thermal niche, with even modest warming expected to degrade its habitat. The reliance of *Ageratina adenophora* on dry season temperature implies that its future spread depends not only on annual warming, but also on changes in seasonal thermal regimes [31]. Human Footprint (HF) acted as a secondary but significant filter, with contribution rates ranging from 11.3% to 22.5%. The unimodal response of all three species to HF—peaking at moderate disturbance levels—indicates that intermediate human activities (e.g., road construction, grazing, land-use change) create open habitats that facilitate weed establishment. However, the optimal HF level differs among species: *Chromolaena odorata* peaks at lower HF (20–40), while *Ageratina adenophora* and *Cirsium japonicum* peak at higher HF (40–50). This suggests that *Chromolaena odorata* is more sensitive to disturbance and may benefit from even low-intensity human activity, whereas the other two species require more pronounced disturbance to achieve similar spread [30,32].

The contrasting future dynamics can be interpreted by distinguishing environmental from anthropogenic causes. *Chromolaena odorata* expands steadily under all scenarios. This reflects its broad thermal tolerance and positive response to moderate disturbance [33]. Climate warming facilitates its dispersal potential, and human-modified landscapes provide corridors for northward spread. Under high emissions, *Ageratina adenophora* contracts in the south but expands in the north. Warming exceeds its dry season optimum in southern lowlands, causing habitat loss while simultaneously opening new suitable areas at higher altitudes where temperature seasonality is lower. This suggests that extreme warming may enable *Ageratina adenophora* to cross altitudinal barriers, a novel finding for montane grassland management. By contrast, the unidirectional habitat loss of *Cirsium japonicum* results from its narrow thermal optimum, high temperature sensitivity, and limited dispersal capacity. Even though its distribution center shifts northward across three prefectures, this migration is too slow to offset the rapid habitat degradation, highlighting the vulnerability of native species with specialized niches.

The limited centroid shifts for the two invasive species indicate that their distributions have already become relatively stable under the current climate, with only minor adjustments to warming. In contrast, the significant centroid migration of *Cirsium japonicum* demonstrates its sensitivity to climate change but also its inadequate capacity to track suitable conditions. Together, these findings underscore that environmental factors define fundamental niches, while anthropogenic disturbance modulates realized distributions. The asymmetric risks—invasive species expanding and native species declining—are driven by the interaction of climate warming with species-specific thermal tolerances and human-altered landscapes. This finding provides refined spatial guidance for formulating regional prevention and control strategies [34].

### 3.3. Differentiation Between Invasive and Native Species and Management Strategies

*Cirsium japonicum* was included in this study based on the actual harm it causes within Yunnan’s grassland ecosystems. Although *Cirsium japonicum* is a native species, it is unpalatable to livestock because it is spinose and readily forms dominant populations in degraded grasslands, severely crowding out desirable forage and making it a signature noxious plant in locally degraded grasslands. Invasive species, leveraging broad ecological amplitudes and strong adaptability, exhibit stable to slightly expanding trends, whereas the native species *Cirsium japonicum* faces significant habitat degradation risk. As a native species, *Cirsium japonicum* also possesses potential ecological value. Its well-developed root system helps to stabilize soil and prevent erosion. Additionally, *Cirsium japonicum* is used in traditional medicine for its heat-clearing, detoxifying, blood-cooling, and hemostatic properties, with its medicinal value widely recognized [35]. Therefore, the management of *Cirsium japonicum* should not simply include eradication approaches. In mildly degraded grasslands, moderate utilization can control its population density while retaining its ecological functions. In moderately to severely degraded areas, measures such as reseeding desirable forages and rotational grazing should be implemented to gradually restore grassland community structure. Notably, *Cirsium japonicum* is sensitive to high temperatures, and future climate change will significantly limit its suitable habitat. Thus, population management must fully consider its dynamic trends to avoid local extinction due to excessive intervention.

For the two invasive species, control strategies should be based on their predicted dynamics and bioecological traits. *Chromolaena odorata* shows stable expansion under all scenarios, with new suitable areas emerging in eastern and northeastern Yunnan. Current control measures in Yunnan grasslands are limited. Manual removal and broad-spectrum herbicides are used only in small, high-value areas due to high costs. Biological control using the gall fly *Procecidochares utilis* has been attempted for *Ageratina Adenophora*, with limited success. Most residents consider these weeds unavoidable, and no large-scale eradication programs exist. Human activities such as road construction, vehicle movement, and livestock grazing inadvertently facilitate seed dispersal, especially for the two invasive species. Early detection and rapid response are recommended along its expansion frontiers, focusing on preventing seed spread (its seeds are wind-dispersed by pappus). Mechanical removal before flowering can reduce seed output. *Ageratina adenophora* exhibits a southern contraction versus northern expansion pattern under high emissions. In southern Yunnan (Xishuangbanna, Puer), where its habitat is naturally declining, control efforts can be relaxed; in northwestern and northeastern Yunnan, where it may breach high-altitude barriers, proactive monitoring and targeted removal are advised. Both invasive species have high seed production and allelopathic effects, so restoring native vegetation cover after removal is essential to prevent re-invasion. As emphasized by López-Tirado and Gonzalez-Andujar (2023) [22], integrating climate-informed species distribution models into national and regional weed management frameworks is essential for adapting agricultural practices to climate change, prioritizing monitoring efforts, and mitigating economic losses in agricultural systems.

### 3.4. Study Limitations

This study has several limitations. First, sampling bias may persist despite spatial thinning. Second, MaxEnt predicts potential habitat suitability but ignores dispersal capacity and vectors; actual colonization depends on these factors, so our results reflect potential rather than realized potential habitat dynamics. Invasive species with high dispersal may still expand as predicted, but native species with limited dispersal could face even greater contraction. Third, the model assumes niche conservatism, ignoring evolutionary change and interspecific interactions [36]. Fourth, Human Footprint data are assumed constant, potentially underestimating future human impacts. Future research should integrate dispersal models and validate predictions with field experiments.

## 4. Materials and Methods

### 4.1. Species Distribution Data

Species distribution data were obtained from the Yunnan Provincial Grassland Pest Survey (2021–2024). Field sampling used 4923 standard plots (1 m × 1 m) established along 2230 transect lines. In each plot, density (individuals/m^2^) was recorded as the number of independent rooted shoots, because none of the three study species (*Chromolaena odorata*, *Ageratina adenophora*, and *Cirsium japonicum*) exhibited persistent clonal connections (e.g., rhizomes or stolons) in the field. When the target species occurred at low to moderate density, all individuals within the entire 1 m × 1 m plot were counted directly. When the target species occurred at such high density that accurate counting of the whole plot was impractical, five 0.1 m × 0.1 m subplots were randomly placed within the plot; the subplot counts were averaged and converted to individuals/m^2^. Cover (%) was visually estimated using a 1 m × 1 m gridded frame (100 cells), averaged by two independent surveyors. Height (cm) was measured as the mean of 10 randomly selected individuals per species per plot. Importance value (IV) was calculated as IV = (relative density + relative cover + relative height)/3, where each relative metric is the species’ value divided by the sum of all species in the plot (×100). After spatial thinning (5 km minimum distance) [37], 1753 occurrence points were retained (296 for *Chromolaena odorata*, 1267 for *Ageratina adenophora*, and 190 for *Cirsium japonicum*) for MaxEnt modeling. Biological and ecological traits were compiled from field observations and the published literature [38,39,40].

### 4.2. Acquisition of Environmental Variable Data

The climatic and environmental variables selected for this study comprised multi-source data, including climate, topography, soil, vegetation, and human activity. A total of 19 bioclimatic factors were sourced from the WorldClim database [41], with a spatial resolution of 30 arc-seconds (approximately 1 km) [42]. Current climate data represented averages for the 1970–2000 period. Future climate data were derived from the BCC-CSM2-MR model developed by the Beijing Climate Center, which has demonstrated good adaptability for simulating climate change in China. Future climate projections covered two periods, 2021–2040 and 2041–2070, representing the near-term (2030s) and mid-term (2050s), respectively, under three Shared Socio-economic Pathways (SSPs): SSP1-2.6 (low emissions), SSP2-4.5 (medium emissions), and SSP5-8.5 (high emissions).

For topographic data, altitude was obtained from the WorldClim database, while aspect and slope were derived from altitude using spatial analysis tools in ArcGIS software. Soil data were obtained from the second edition of the China Soil Characteristics Dataset released by the School of Atmospheric Sciences, Sun Yat-sen University [43]. In this study, soil properties were categorized into two layers: topsoil (T_, 0–30 cm) and subsoil (S_, 30–60 cm). Normalized Difference Vegetation Index (NDVI) data were obtained from the monthly NDVI dataset for China at 1 km resolution, provided by the National Earth System Science Data Center [44].

Human Footprint data were obtained from the global terrestrial Human Footprint dataset published by Mu et al. [32]. This dataset integrates eight anthropogenic pressure indicators: built environments, population density, nighttime lights, croplands, pastures, roads, railways, and navigable waterways. It generates an annual Human Footprint index at 1 km resolution through a standardized scoring system, effectively reflecting the intensity and spatiotemporal dynamics of human pressure on natural ecosystems.

### 4.3. Processing and Selection of Environmental Variables

To avoid multicollinearity, spatial autocorrelation, and model overfitting among environmental variables, we systematically screened the candidate environmental factors. First, all candidate variables were input into the MaxEnt model along with the species distribution data for a preliminary run to obtain each variable’s contribution rate. Concurrently, environmental variable values were extracted from 10,000 randomly generated sampling points within the study area, and Pearson correlation coefficients were calculated to assess correlations between variables (|r| > 0.8 considered highly correlated). Integrating the contribution rates and correlation analysis results, variables were retained if they met the criteria of a correlation coefficient |r| < 0.8 and a contribution rate greater than 1%, thereby eliminating highly correlated variables and those with negligible contributions. This process yielded specific sets of environmental factors for each species [45]. For *Chromolaena odorata*, 13 variables were retained, including 7 bioclimatic variables, 3 soil variables, 1 topographic variable, the Human Footprint variable, and the NDVI variable. For *Ageratina adenophora*, 12 variables were retained, including 5 bioclimatic variables, 3 soil variables, 2 topographic variables, the Human Footprint variable, and the NDVI variable. For *Cirsium japonicum*, 10 variables were retained, including 6 bioclimatic variables, 2 soil variables, the Human Footprint variable, and the NDVI variable.

### 4.4. MaxEnt Parameter Optimization and Model Construction

To optimize the predictive performance of the Maximum Entropy (MaxEnt) model, we systematically tuned the regularization multiplier (RM) and feature class (FC) using the ENMeval package (version 2.0.4) [46]. Based on the selected environmental variables and species distribution data, candidate RM values were set from 0.5 to 4.0, with a step size of 0.5. Candidate FC combinations included L (linear), LQ (linear + quadratic), H (hinge), LQH (linear + quadratic + hinge), LQHP (linear + quadratic + hinge + product), and LQHPT (linear + quadratic + hinge + product + threshold), resulting in a total of 6 feature combinations and 48 candidate models. The “block” partitioning method was used to divide the study area into 4 spatial blocks. For each iteration, 3 blocks were used as the training set and 1 block as the validation set for cross-validation, repeated 10 times to assess model stability. Model runs were performed using the Java implementation of MaxEnt (version 3.4.4, maxent.jar), with 10,000 randomly sampled background points. The corrected Akaike Information Criterion (AICc) was used as the primary evaluation metric, in conjunction with the training AUC (auc.train) and the average validation AUC (auc.val.avg). The model with the lowest AICc value was selected as the optimal parameter combination for subsequent species habitat suitability predictions [47].

### 4.5. Current Habitat Suitability Prediction

Using the optimized MaxEnt model parameters, the selected species distribution data and environmental variables were input into the model for computation. During model runs, 75% of the occurrence points were randomly selected as the training set, and the remaining 25% were used as the validation set. The number of background points was set to 10,000, and the maximum number of iterations was set to 5000. The model was run with 10 replicates to reduce random errors. Model predictive accuracy was evaluated using the Area Under the Curve (AUC) of the Receiver Operating Characteristic (ROC) curve. AUC values closer to 1 indicate stronger predictive ability: 0.5–0.7 is considered fair, 0.7–0.9 is considered good, and above 0.9 is considered excellent [48]. The raster output from the model represented species occurrence probability (0–1). Visualization and reclassification were performed using ArcGIS 10.8 software, dividing suitability levels into four categories: unsuitable (0–0.1), low-suitability (0.1–0.3), medium-suitability (0.3–0.5), and high-suitability (0.5–1). The area of each suitability class was calculated.

### 4.6. Spatiotemporal Dynamics of Suitable Habitats

To reveal the spatiotemporal evolution characteristics of species’ suitable habitats under future climate scenarios, spatial overlay analysis was conducted on the prediction results for the 2050s and 2070s under the SSP1-2.6, SSP2-4.5, and SSP5-8.5 scenarios, using the current climate habitat distribution as a baseline. First, raster data for each period were reclassified into suitable (>0.1) and unsuitable (≤0.1) areas and converted to vector format. Overlay analysis was performed using spatial analysis tools in ArcGIS 10.8 to classify future changes in suitable areas relative to the current distribution into three types: stable areas (suitable both currently and in the future), contraction areas (currently suitable but becoming unsuitable in the future), and expansion areas (currently unsuitable but becoming suitable in the future). The area and spatial distribution characteristics of each change type were calculated.

### 4.7. Average Center-Point Shift

The Mean Center analysis method was employed to investigate the shift trajectories of the species’ distribution centers under future climate scenarios. Based on the high-suitability raster data for the current period and for each scenario in the 2050s and 2070s, the “Raster to Polygon” tool in ArcGIS 10.8 was used to convert the data into vector polygon features, extracting the spatial extent of high-suitability areas for each period. The “Mean Center” tool was then used to calculate the coordinates of the distribution centers for the current period and for each future scenario. Subsequently, the “Points to line” tool was used to connect the center points in chronological order, generating trajectory maps of the shifts. By calculating the straight-line distance and azimuth between center points of adjacent periods, the direction and intensity of distribution center shifts under different climate scenarios were quantitatively analyzed.

## 5. Conclusions

This study reveals three novel ecological patterns regarding the divergent responses of invasive and native Asteraceae weeds to climate change in Yunnan grasslands.

First, the three species are driven by fundamentally different thermal response curves. *Chromolaena odorata* shows a sustained positive response to mean annual temperature (contribution 67.6%), indicating that further warming will continuously expand its suitable habitat. In contrast, the native *Cirsium japonicum* exhibits a strictly unimodal response with a narrow optimum at 0–10 °C (contribution 46.4%), meaning that even modest warming will degrade its habitat. *Ageratina adenophora* is primarily limited by dry season temperature (contribution 48.5%), with an optimum range of 10–15 °C.

Second, future potential habitat dynamics are asymmetrical. Under all emission scenarios, *Chromolaena odorata* expands eastward and northward, with contraction areas approaching zero. *Ageratina adenophora* contracts in southwestern Yunnan under low/medium emissions but switches to a southern contraction versus northern expansion pattern under high emissions, suggesting that extreme warming may enable it to cross high-altitude barriers. By contrast, *Cirsium japonicum* undergoes unidirectional habitat loss, with over 50% of its current suitable area projected to disappear by the 2050s under high emissions.

Third, the native species exhibit stronger climate sensitivity but limited migration capacity. Its distribution center shifts across three prefectures (Lijiang, Dali, Chuxiong)—much farther than those of the invasive species (Kunming, Yuxi, Chuxiong)—yet this migration is insufficient to offset the rate of habitat loss.

These findings establish a quantitative, trait-based framework for predicting invasive species expansion and native species decline under climate change. They also provide a mechanistic basis for differentiating management strategies: proactive control along expansion frontiers for invasive species versus targeted conservation in climatic refugia for native medicinal species.

## Figures and Tables

**Table 1:** Comparison of biological and ecological characteristics of *Ageratina adenophora*, *Chromolaena odorata*, and *Cirsium japonicum*.

Characteristic	*Ageratina adenophora* (Invasive)	*Chromolaena odorata* (Invasive)	*Cirsium japonicum* (Native)
Importance value	31.5	24.2	8.2
Density (plants/m^2^)	18.6 ± 12.3	15.2 ± 10.5	2.5 ± 1.9
Cover (%)	32.5 ± 18.6	26.3 ± 14.2	7.2 ± 4.3
Height (cm)	68.4 ± 31.2	82.1 ± 35.6	48.6 ± 22.4
Life form	Perennial subshrub	Perennial shrub	Perennial herb
Life history and reproduction	Sexual + vegetative (stem fragments, root buds); very high seed output (30,000–150,000 seeds/plant); dispersal by wind (pappus), water, animals (fur), and human activities	Sexual + vegetative (stem fragments); very high seed output; dispersal by wind (pappus), water, and human activities	Primarily sexual; moderate seed output; dispersal by wind (pappus, short-distance) and animals (spiny involucre attaches to fur/clothing)
Niche and adaptability	Broad niche; tolerates diverse soils and light conditions; positive response to moderate disturbance; moderately drought-tolerant, cold-sensitive	Relatively broad niche; prefers sandy, infertile soils; light-demanding; drought-tolerant, fire-resistant, cold-sensitive	Moderate niche; prefers clay-rich, moist soils; high temperature sensitivity; low drought tolerance
Competition and allelopathy	Strong allelopathy (root exudates, litter); resource competition; forms monodominant stands	Strong allelopathy (leaf extracts); vertical space competition (height advantage); forms dense thickets	Weak or no allelopathy; limited competitive ability; typically subordinate in communities
Habitat preference	Warm (10–22 °C), moderately disturbed, clay-rich soils	Warm (>18 °C), humid lowland valleys, sandy soils	Cool-temperate (10–18 °C), moist, clay-rich fertile soils

## Data Availability

The census data is stored in the Forestry and Grassland Bureau of Yunnan Province, which is not open to the public. If there are special needs, we can ask the author for this part of the data used in the paper.
